# Cervical cancer mortality among young women in Latin America and the Caribbean: trend analysis from 1997 to 2030

**DOI:** 10.1186/s12889-021-12413-0

**Published:** 2022-01-16

**Authors:** J. Smith Torres-Roman, Luz Ronceros-Cardenas, Bryan Valcarcel, Janina Bazalar-Palacios, Jorge Ybaseta-Medina, Greta Carioli, Carlo La Vecchia, Christian S. Alvarez

**Affiliations:** 1 Universidad Científica del Sur, Lima, Peru; 2 Latin American Network for Cancer Research (LAN−CANCER), Lima, Peru; 3 Universidad Católica Los Ángeles de Chimbote, Instituto de Investigación, Chimbote, Peru; 4 Escuela de Medicina Humana, Universidad Nacional San Luis Gonzaga, Ica, Peru; 5 Department of Clinical Sciences and Community Health, Università degli Studi di Milano, 20133 Milan, Italy

**Keywords:** Cervical neoplasm, Latin America and the Caribbean, Trends, Mortality, Forecast

## Abstract

**Background:**

Cervical cancer continues to show a high burden among young women worldwide, particularly in low- and middle-income countries. Limited data is available describing cervical cancer mortality among young women in Latin America and the Caribbean (LAC). The purpose of this study was to examine the mortality trends of cervical cancer among young women in LAC and predict mortality rates to 2030.

**Methods:**

Deaths from cervical cancer were obtained from the World Health Organization mortality database. Age-standardized mortality rates per 100,000 women-years were estimated in women aged 20–44 years using the world standard population for 16 countries (and territories) in LAC from 1997 to 2017. We estimated the average mortality rates for the last 4 years (2014–2017). Joinpoint regression models were used to identify significant changes in mortality trends. Nordpred method was used for the prediction of the mortality rates to 2030.

**Results:**

Between 2014 and 2017, Paraguay and Venezuela had the highest mortality rates of cervical cancer, whereas Puerto Rico had the lowest rates. Overall, most of the LAC countries showed downward trends of cervical cancer mortality over the entire period. Significant decreases were observed in Chile (Average annual percent change [AAPC]: − 2.4%), Colombia (AAPC: − 2.0%), Cuba (AAPC: − 3.6%), El Salvador (AAPC: − 3.1%), Mexico (AAPC: − 3.9%), Nicaragua (AAPC: − 1.7%), Panama (AAPC: − 1.7%), and Peru (AAPC: − 2.2%). In contrast, Brazil (AAPC: + 0.8%) and Paraguay (AAPC: + 3.7%) showed significant upward trends. By 2030, mortality rates are not predicted to further decrease in some LAC countries, including Argentina, Paraguay, and Venezuela.

**Conclusions:**

Mortality trends of cervical cancer among young women have large variability in LAC countries. Cervical cancer screening programs have a high priority for the region. Primary and secondary prevention in the community are necessary to accelerate a reduction of cervical cancer mortality by 2030.

**Supplementary Information:**

The online version contains supplementary material available at 10.1186/s12889-021-12413-0.

## Background

Cervical cancer remains a major public health problem in low- and middle-income countries (LMICs) [1–3]. In 2020, GLOBOCAN estimated 604,000 new cases and 342,000 deaths from cervical cancer worldwide, with 80% occurring in LMICs [3], mainly sub-Saharan Africa, South-Eastern Asia and Latin America and the Caribbean (LAC). Although substantial declines in incidence rates have been observed worldwide, particularly in European countries [4, 5], cervical cancer continues to affect disproportionately women in LAC compared with most other regions [6].

Due to the high burden of this disease, in 2020 the World Health Organization (WHO) adopted a global strategy to decrease the number of new cervical cancer cases, with the aim of maintaining an incidence rate below 4 per 100,000 women [7]. Currently, 29 of the 47 countries (and territories) in the LAC region have implemented vaccination programs for girls and almost all countries have screening services in place, and while certain screening programs in the region achieve considerable coverage [8, 9], the ambitious goals set by the cervical cancer elimination strategy [10] have yet to be achieved. Despite advances in treatment in the LAC region, barriers to treatment access persist.

Cervical cancer continues to be one of the main cancers, and affecting women under 45 years of age [1]. Although previous studies have reported a remarkable decline of cervical cancer mortality among young women in several European countries [4, 5], LMICs report an increase in cases and deaths in women under 45 years of age [2, 6, 11]. This rapid rise of cases of cervical cancer among young women could be explained, in part, by changes in sexual behaviors (e.g. early sexual activity) and a subsequent increase in the risk of human papillomavirus (HPV) infection [12–14]. In fact, the incidence of HPV infection in Latin America is higher compared to the average worldwide, [14] being attributable to more than 50,000 new cases of cervical cancer per year [14]. Nowadays, many LMICs have not introduced the HPV vaccine into their national immunization schedules [7]. Among countries that offer the HPV vaccine in this region, the coverage varies from 30% in Uruguay to 81% in Panama for the full dose schedule (2–3 doses) in girls aged 14–15 years [15, 16]. Furthermore, early detection programs for precancerous cervical lesions have not had an impact in this region compared to developed countries [17].

To our knowledge, mortality rates and trends of cervical cancer in young women have not been evaluated in LAC. Therefore, we examined mortality trends of cervical cancer among young women (aged 20–44 years) from 16 LAC countries (and territories) from 1997 to 2017. In addition, we projected cervical cancer mortality rates to 2030 and analyzed the changes according to the risk and demographic components.

## Methods

### Data source

Data were obtained from the WHO Mortality Database from 1997 (or the first year available) to 2017 (or the last year available) from 16 LAC countries. Bolivia, Guatemala, and Honduras were excluded because data were not available for more than 5 years. According to the 10th revision of the International Classification of Diseases (ICD-10), which was used by all countries at the time of the study, we identified cervical neoplasm by code C53 (malignant neoplasm of cervix uteri). Population figures were obtained from the United Nations World Population Prospects 2017 Revision, by age, country and year (up to 2030) [18].

### Statistical analysis

Age-standardized mortality rate (ASMR) for each 5-year age group (i.e., 20–24, 25–29, 30–34, 35–39, 40–44 years) and 21 calendar years were computed and adjusted using the word standard population per 100,000 women-years [19]. We estimated the average mortality rates for the last 4 years (2014–2017) to show current rates in the region.

Joinpoint regression analysis was performed to examine the mortality trends using the Joinpoint Regression Program version 4.7.0 [20]. For each of the trends identified by the Joinpoint model, we computed the estimated annual percent changes (APC) and the average annual percent change (AAPC) over the whole period. The significance levels used were based on the Monte Carlo permutation method, using the logarithm of the ratio [20, 21]. We considered *p*-values < 0.05 as statistically significant.

To predict the mortality rates to 2030, we used the Nordpred package in R software based on an age-period-cohort model (5-year calendar periods and 5 age groups). For Venezuela, the year 2014 was taken as a reference due to the lack of available information. The predictions of the most recent linear trend for the last fithteen years were attenuated in the drift parameter of 0% in the first period, 50% in the second period, and 50% from the third prediction period [22]. The objective of this mathematical operation is to reduce the influence of the current trend on predictions. The proposed model is based on empirical comparisons from different methods of predictions [22].

We also computed the predicted mortality changes from 2015 to 2030 according to the respective risk and demographic change components. These two components can be different from zero and can be either positive or negative in direction. The formula is expressed as (26)

$$\Delta \mathrm{tot}=\Delta \mathrm{risk}+\Delta\ \mathrm{pop}=\left(\mathrm{Nfff}-\mathrm{Noff}\right)+\left(\mathrm{Noff}-\mathrm{Nooo}\right)$$

Where Δtot is the total change, Δrisk is the change in function of risk, Δpop is the change in function of the population, Nooo is the number of observed cases, Nfff is the number of projected cases, and Noff is the number of expected cases when the mortality rates increase during the observed period.

In addition to the main analysis, given that cancer of the corpus uteri is rare among women below the age of 50 and they are commonly assigned to cervical cancer deaths, deaths coded as “malignant neoplasm of corpus uteri” (C54 in ICD-10) as well as deaths coded as “malignant neoplasm of uterus, part not specified” (C55 in ICD-10), were assigned to cervical cancer mortality and we reran the Joinpoint Regression models and predicted the mortality rates. The results of these analyses are presented in the Supplementary Materials.

## Results

**Fig. 1:**
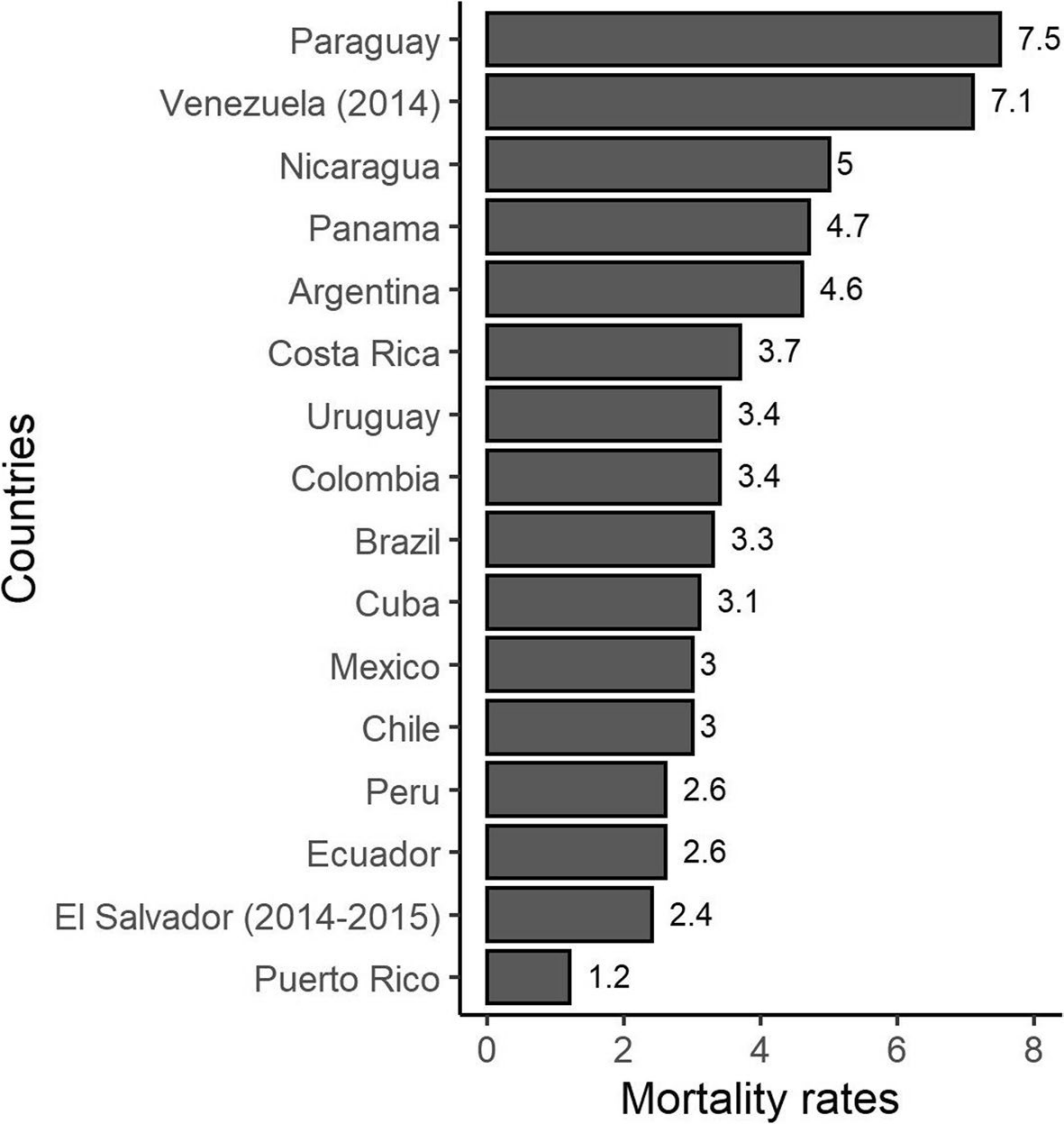
Age-standardized (world population) mortality rates of cervical cancer per 100,000 women aged 20–44 years in Latin American and Caribbean countries from 2014 to 2017 (unless specified in parentheses)

Figure 1 shows the average age-standardized mortality rates (ASMR) per 100,000 women-years of cervical cancer among young women in LAC countries from 2014 to 2017. The highest ASMR were observed in Paraguay and Venezuela (above 7 per 100,000 women-years), while Puerto Rico had the lowest rate (1.2 per 100,000 women-years).

Table 1 shows the number of deaths due to cervical cancer, cancer of the uterine corpus (corpus) and unspecified uterine cancer. In 1997, the percentage of deaths from corpus cancer ranged from 0% in Paraguay, Nicaragua, and Uruguay to 20% in Puerto Rico, whereas in the last year of the observation ranged from 0% in Nicaragua to 21.4% in Puerto Rico. In 1998, deaths from unspecified uterine cancer ranged from 3.9% in Chile to 50% in El Salvador, whereas in the last year of observation, the percentage ranged from 0% in Costa Rica to 29.1% in Ecuador.

**Table 1:** Number of deaths due to cervical cancer, corpus cancer and unspecified uterine cancer among women aged 20–44 years in Latin America and the Caribbean, 1997^a^ and 2017^b^

Country	Number of deaths in 1997^a^			Number of deaths in 2017^b^		
	Cervix	Corpus	unspecified Uterine (%)	Cervix	Corpus	unspecified Uterine (%)
Argentina	308	22(4.6%)	152(31.5%)	410	12(2.1%)	155(26.9%)
Brazil	925	28(2.1%)	370(27.9%)	1634	73(3.7%)	253(12.9%)
Chile	171	1(0.6%)	7(3.9%)	108	4(3.2%)	14(11.1%)
Colombia	321	8(2.1%)	60(15.4%)	351	12(3%)	33(8.3%)
Costa Rica	33	2(5.4%)	2(5.4%)	37	2(5.1%)	0(0%)
Cuba	118	32(16.8%)	41(21.5%)	76	18(18%)	6(6%)
Ecuador	57	2(1.7%)	56(48.7%)	82	8(6.3%)	37(29.1%)
El Salvador	26	5(8.1%)	31(50%)	27	2(5.9%)	5(14.7%)
Mexico	948	20(1.9%)	76(7.2%)	794	68(7.4%)	57(6.2%)
Nicaragua	63	0(0%)	5(7.4%)	62	0(0%)	1(1.6%)
Panama	32	2(5.3%)	4(10.5%)	40	1(2.3%)	2(4.7%)
Paraguay	35	0(0%)	42(54.5%)	104	3(2.4%)	17(13.7%)
Peru	164	3(1.3%)	66(28.3%)	160	10(4.8%)	38(18.3%)
Puerto Rico	6	2(20%)	2(20%)	7	3(21.4%)	4(28.6%)
Uruguay	23	0(0%)	9(28.1%)	24	2(5.7%)	9(25.7%)
Venezuela	294	5(1.3%)	94(23.9%)	411	13(2.8%)	39(8.4%)

^a^1997 or first available year after 1997

^b^2017 or last available year before 2017

Table 2 displays the Joinpoint analysis of cervical cancer mortality in LAC countries. Overall, most countries showed a significant decrease in cervical cancer mortality from 1997 to 2017: Chile (AAPC: −2.4%; 95% CI: -3.7, -1.1), Colombia (AAPC: − 2.0%; 95% CI: − 2.6, − 1.4), Cuba (AAPC: − 3.6%; 95% CI: − 4.9, − 2.3), El Salvador (AAPC: − 3.1%; 95% CI: − 5.2, − 1.0), Mexico (AAPC: − 3.9%; 95% CI: − 4.6, − 3.3), Peru (AAPC: − 2.2%; 95% CI: − 3.5, − 0.9), Nicaragua (AAPC: − 1.7%; 95% CI: − 2.8, − 0.6) and Panama (AAPC: − 1.7%; 95% CI: − 3.2, − 0.2). In contrast, Paraguay (AAPC: 3.7%; 95% CI: 2.5, 4.8) and Brazil (AAPC: 0.8%; 95% CI: 0.3, 1.3) showed significant increases in cervical cancer mortality for the entire period. In addition, significant downward trends during the first years of evaluation were observed in Argentina, Brazil, Chile and Costa Rica, with subsequent upward trends during the last period.

**Table 2:** Joinpoint analysis for cervical cancer mortality in Latin America and the Caribbean, 1997-2017

Countries	Year	APC	95%CI	Year	APC	95%CI	AAPC	95%CI
Argentina	1997–2007	−2.8*	− 4.2, − 1.4	2007–2017	3.5*	2.0, 4.9	0.3	− 0.7, 1.2
Brazil	1997–2007	− 0.9*	− 1.6, − 0.2	2007–2017	2.5*	1.8, 3.2	0.8*	0.3, 1.3
Chile	1997–2011	−5.4*	− 6.5, − 4.3	2011–2017	4.8*	0.6, 9.3	−2.4*	− 3.7, − 1.1
Colombia	1997–2017	−2.0*	− 2.6, − 1.4				−2.0*	− 2.6, − 1.4
Costa Rica	1997–2006	−6.2*	− 10.5, − 1.8	2006–2017	3.5*	0.1, 7.1	−1.0	− 3.6, 1.6
Cuba	1997–2017	−3.6*	− 4.9, − 2.3				−3.6*	− 4.9, − 2.3
Ecuador	1997–2017	−0.1	−1.5, 1.3				−0.1	−1.5, 1.3
El Salvador	1997–2015	−3.1*	− 5.2, − 1.0				−3.1*	− 5.2, − 1.0
Mexico	1997–2017	−3.9*	− 4.6, − 3.3				−3.9*	− 4.6, − 3.3
Nicaragua	1997–2017	−1.7*	− 2.8, − 0.6				−1.7*	− 2.8, − 0.6
Panama	1997–2017	−1.7*	− 3.2, − 0.2				−1.7*	− 3.2, − 0.2
Paraguay	1997–2017	3.7*	2.5, 4.8				3.7*	2.5, 4.8
Peru	1997–2017	−2.2*	− 3.5, − 0.9				− 2.2*	− 3.5, − 0.9
Puerto Rico	1999–2017	2.5	− 3.3, 8.5				2.5	−3.3, 8.5
Uruguay	1997–2017	−1.2	−6.3, 4.1				−1.2	−6.3, 4.1
Venezuela	1997–2014	0.5	−0.3, 1.2				0.5	−0.3, 1.2

*significantly different from 0 (*p* < 0.05)

**Fig. 2:**
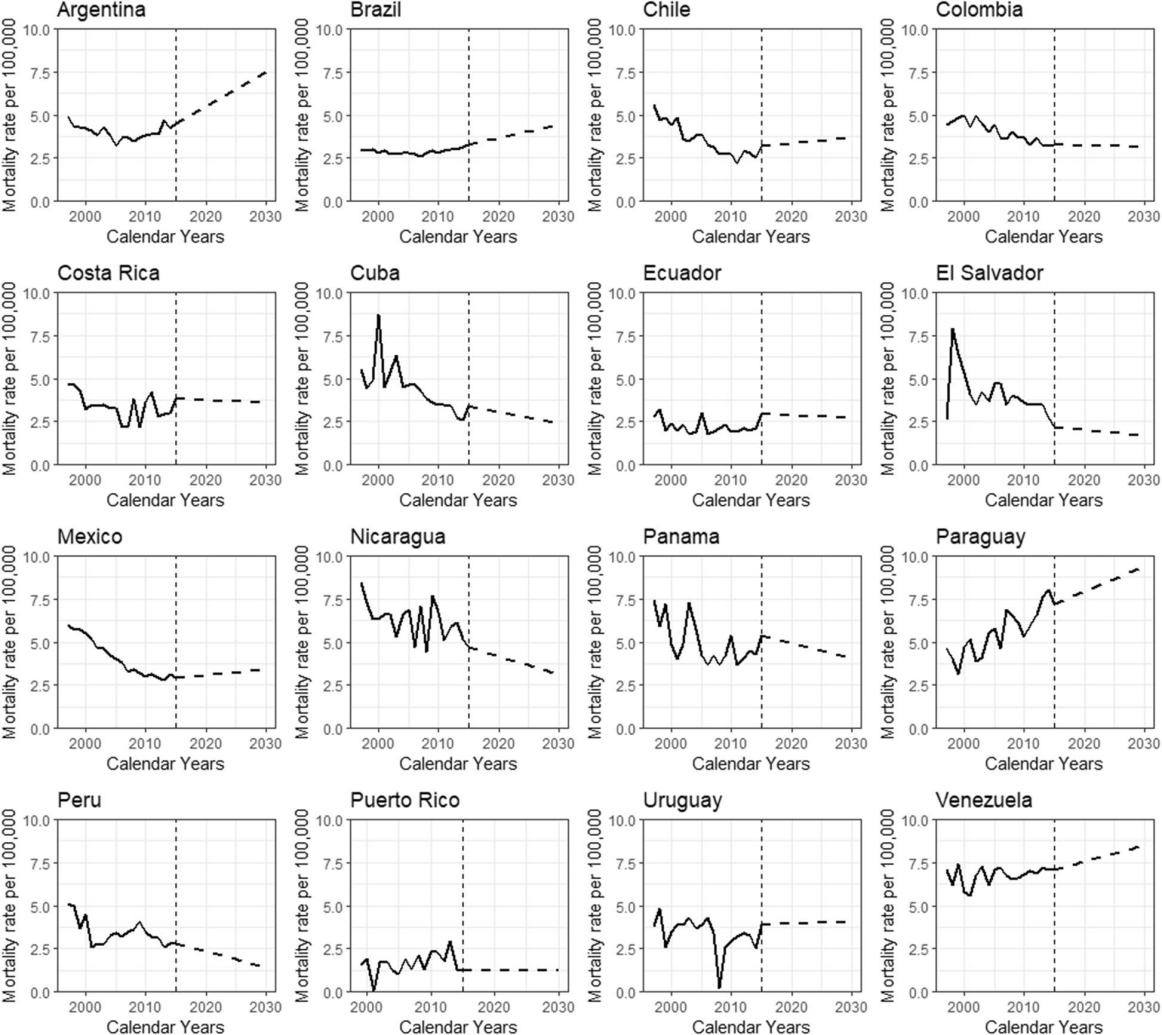
Age-standardized (world population) mortality trends of cervical cancer from 1997 to 2017 and predicted mortality rates through 2030 in Latin America and the Caribbean

Figure 2 illustrates cervical cancer mortality trends from 1997 to 2030 for 16 LAC countries. Argentina, Brazil, Paraguay, and Venezuela showed continuous upward trends to 2030, whereas Nicaragua, Panama, and Peru presented downward trends to 2030.

**Fig. 3:**
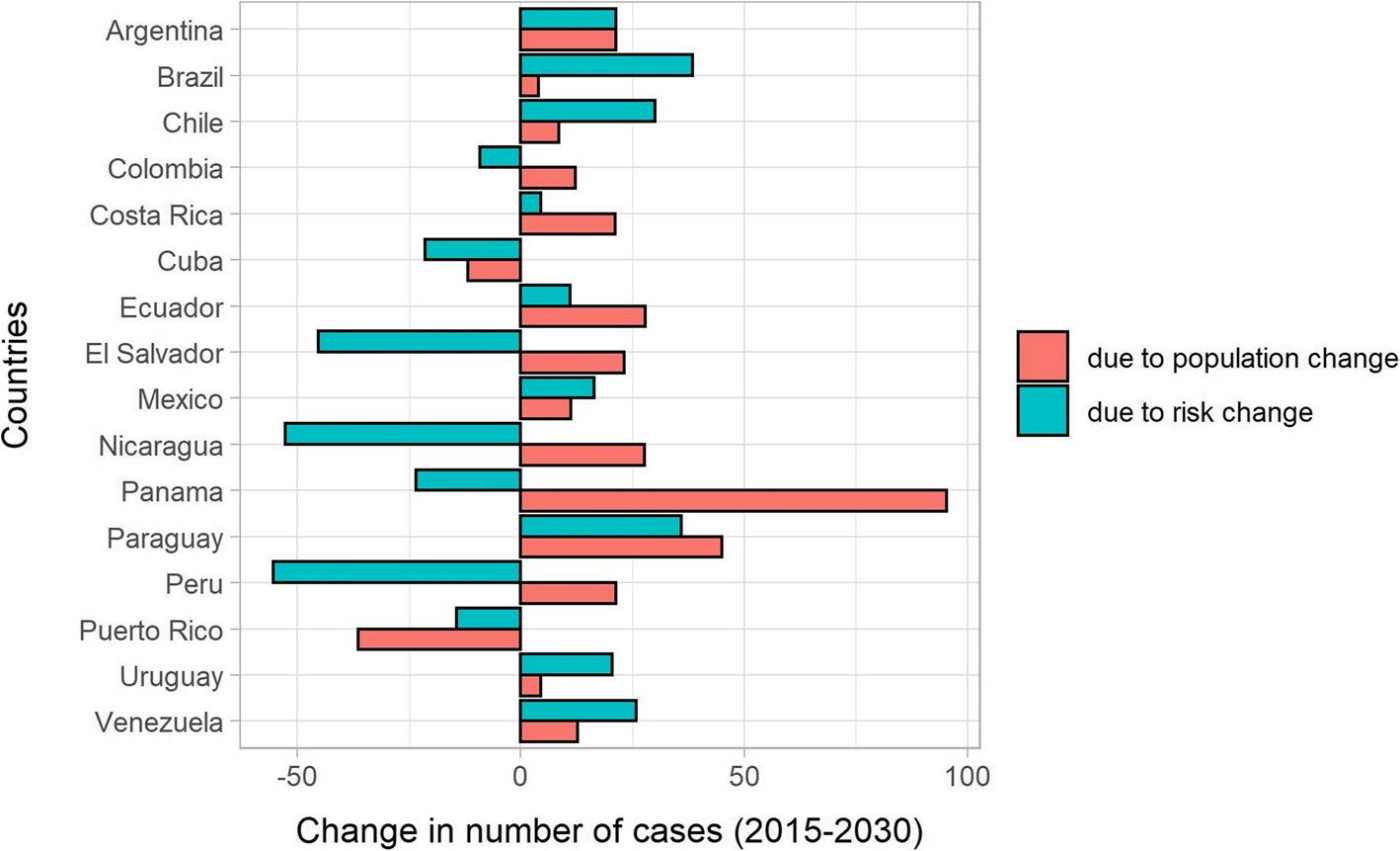
Cervical cancer mortality in Latin America and the Caribbean for 2015 and 2030, total change (change), change due to change in risk (risk), and change due to demographic alterations (population)

Table 3 and Fig. 3 show the number of cervical cancer deaths, ASMR and percentage change in cases due to population and risk between 2015 and 2030. As the predicted number of new cervical cancer cases in 2030 in a given country reflects both changing rates and national population projections, the countries with the largest populations proportionally have the largest number of future cervical cancer patients. For example, Colombia, El Salvador, and Panama had the greatest increase in population compared to change due to risk, which was negative, resulting in an overall increase.

**Table 3:** Number of cervical cancer deaths, age-standardized mortality rates, and percentage change in cases due to population and risk among women aged 20–44 in Latin America and the Caribbean, 2015 and 2030

Country	Population (annual million)		Number of deaths		Age-standardized rates		Total change (%)	Change due to population (%)	Change due to risk (%)
	2015	2030	2015	2030	2015	2030			
Argentina	7.9	8.6	372	695	4.5	7.5	82.4	21.3	61.1
Brazil	41.3	40.5	1430	2030	3.2	4.4	42.5	4.0	38.5
Chile	3.3	3.3	´112	144	3.2	3.7	38.4	8.5	30.0
Colombia	9.5	10.0	321	339	3.3	3.1	3.0	12.2	−9.2
Costa Rica	0.9	1.0	35	41	3.8	3.6	25.8	21.2	4.5
Cuba	1.9	1.7	79	46	3.4	2.4	−33.8	−11.9	−21.4
Ecuador	3.0	3.6	88	104	2.9	2.7	38.7	27.8	11.0
El Salvador	1.2	1.4	27	25	2.2	1.7	−22.2	23.1	−45.3
Mexico	25.0	26.5	756	981	3.0	3.4	27.7	11.2	16.4
Nicaragua	1.2	1.4	56	46	4.6	3.1	−25.0	27.7	−52.7
Panama	0.8	0.9	42	62	5.4	4.1	71.9	95.3	−23.4
Paraguay	1.2	1.5	81	152	7.2	9.4	81.0	45.1	35.9
Peru	6.1	6.6	169	103	2.8	1.4	−34.1	21.3	−55.5
Puerto Rico	0.7	0.5	9	8	1.2	1.2	−50.9	−36.5	−14.4
Uruguay	0.6	0.6	25	27	3.9	4.1	25.0	4.5	20.5
Venezuela	5.8	6.3	411	569	8.1	8.5	38.6	12.8	25.8

As for the sensitivity analysis, we grouped deaths from cervical cancer and cancer of the corpus uteri and uterus unspecified (all of this as cervical cancer, see Supplementary Materials). In the last 4 years, Paraguay and Venezuela had the highest mortality rates, whereas El Salvador and Puerto Rico had the lowest mortality rates. The trends were very similar to the mortality estimates using only cervical cancer deaths code C53. Furthermore, Argentina, Brazil, Chile, and Costa Rica had significant upward trends in the last years, and the remaining countries presented trends similar to the main analysis. As for the predictions to 2030, we observed an increase in cervical cancer deaths in some countries, mainly due to changes in population structure and size.

## Discussion

We evaluated the mortality trends of cervical cancer among young women from 16 LAC countries (and territories) and predicted mortality rates until 2030. Downward trends were observed in Chile, Colombia, Cuba, El Salvador, Mexico, Nicaragua, Panama, and Peru for the entire period; whereas Brazil, Argentina, Chile, and Costa Rica showed initial downward trends from − 0.9% to − 6.2% followed by significant upward trends from 2.5 to 4.8% (for the period~ 2006–2017). In the last 4 years of study (2014–2017), Paraguay and Venezuela had the highest mortality rates, whereas Puerto Rico had the lowest mortality. By 2030, we projected that mortality for cervical cancer will increase in some countries that were examined.

We considered cervical cancer mortality in young women only because of the lack of research in the LAC region, which has focused on cervical cancer mortality in general. In addition, another reason was due to the increase in mortality among young women reported in a number of studies [23, 24]. Because HPV infection is the most important risk factor for cervical cancer, younger women are likely to have experienced higher rates of HPV infection compared to older women.

Cervical cancer mortality rates among young women in LAC are higher than in Eastern European countries [1]. In 2017, Western and Southern European countries reported rates below 2 deaths per 100,000, while Northern and Eastern Europe reported rates below 6 deaths per 100,000 [5]. In Latin America and the Caribbean, the HPV infection is associated with an increased number of sexual partners, which is problematic, especially among young women [17]. Despite the use of conventional cervico-vaginal cytology that has successfully reduced cervical cancer mortality in developed countries [25, 26], this has not been the case among women in LAC. Furthermore, the reduction in cervical cancer mortality that can be achieved through a screening program depends on several factors. For example, the epidemiology of HPV infection in the population and the characteristics of the screening program, including sensitivity and specificity, access to treatment by those in whom lesions are detected and quality of follow-up [27]. Some studies in the region have reported a sensitivity of the cytology between 30–40% [28, 29]. These results were obtained under study conditions and not in real life situations, where perhaps the performance of cytology is even more unsatisfactory. Moreover, screening programs for early detection of precancerous cervical lesions will have no impact in young women if they are not accompanied by an adequate system for the evaluation of positive cases, as well as resources to ensure the treatment of those cases.

Between 2014 and 2017, Paraguay, and Venezuela reported mortality rates above 7 per 100,000, and the rest of countries showed mortality rates above 2 per 100,000 (except for Puerto Rico). One of the potential reasons could be explained by the high prevalence of HPV, predominantly high-risk serotypes (HPV-16 and HPV-18), in LAC countries [14, 30–33]. For instance, some studies have reported a prevalence of high-risk HPV of 71.5% in Paraguay [34], and 95% in Venezuela [35] among women with cervical cancer lesions. In addition, these countries have one of the highest HPV incidence (more than 10 per 100 000 person-years), worldwide [14]. This may explain the mortality observed in these countries; for example, in Paraguay, between 2010 and 2014, cervical cancer was the leading cause of cancer mortality among women in more than half of its health regions [36]. In addition, the national cytological screening program in Paraguay has had a low coverage in recent years (less than 20%) [37]. While in Venezuela, this neoplasm represents the leading cause of mortality among women [38, 39]. In addition, a predominance of abnormal cytological results in women has been reported in Venezuela [39]. All these could explain their higher mortality rates of cervical cancer in these countries.

Other factors that could increase the mortality of cervical cancer in LAC are social inequalities, low-income settings, and difficulty in accessing prompt and adequate health care delivery [40–42]. Despite the contributions of primary health care in expanding the coverage and supply of cervical cancer detection and control in LAC [43, 44], several studies have observed less coverage among women with greater social and economic vulnerability in LAC [45, 46].

On the other hand, the decreasing mortality rates observed in most LAC countries can be related to the development and implementation of public health programs, and community interventions against cervical cancer during the last years [47]. Effective detection of early-stage cervical cancer, followed by optimal treatment, could also explain the reduction in cervical cancer mortality.

Surprisingly, our assessment showed that Argentina, Brazil, Chile, and Costa Rica had an initial downward trend followed by a significant upward trend. It is unclear what is driving the increased mortality of cervical cancer in these countries during the last period of observation. A potential explanation for this finding is likely due to an improvement of cancer-related death certification registry, providing better identification of deaths [48]. Therefore, the increase in mortality could be explained simply as an artifact. However, there is still a need to expand the coverage of cancer registries to obtain more reliable data in LAC to evaluate the outcomes of the interventions carried out within each country.

The variability of cervical cancer mortality rates is substantial in the LAC countries considered in this study. By 2030, mortality will increase in a number of LAC countries, likely due to changes in population size and structure. This underscores the persistent importance of preventing these malignancies through the implementation and reinforcement of targeted public health programs in the region for young women. In recent years, progress has been made in the implementation of policies, detection programs, and interventions for cervical cancer in most of the countries in the Americas [47]. Moreover, the main objective of the WHO is to reduce cervical cancer mortality by 2030 by adopting the following schemes: 90% of girls will be fully vaccinated against HPV by age 15 years, 70% of women will be screened with a high-precision HPV test at 35 and 45 years of age, and 90% of women identified with cervical disease will receive treatment and care [7].

The implementation of vaccination programs against HPV is important to reduce the incidence and mortality of cervical cancer [9, 49], and although many LAC countries have started free vaccination programs aimed at women between the ages of 9 and 13 years in schools and health facilities or health centers [16], vaccination coverage is very low [15]. For example, in 2014, 33% of women aged 10–20 years in developed countries received the full course of the vaccine, compared with 2.7% of women in developing countries [50]. Potential reasons are parental adherence to allow their daughters to be vaccinated against HPV due to limited knowledge of HPV, misguided safety concerns, cost to constrained health systems, and cultural barriers, which is key to the access in children [51–53]. HPV testing has been recommended only for women over the age of 30, since HPV infection often disappears without clinical consequences [54]. Cervical cancer screening programs have shown to be effective in reducing mortality by cervical cancer [55]. However, timely administration of HPV vaccine and decentralizing treatment for cervical cancer are factors that still represent an important challenge to address in LAC.

This study analyzed deaths from uterus cancers regardless of their location (cervix and corpus separately), because of the difficulty to determine exact trends in cervical and uterine corpus cancer mortality [56, 57]. For example, in 1997, Argentina, Brazil, Ecuador, El Salvador, Paraguay, Peru and Uruguay had more than 25% of unspecified uterine cancer deaths, and in 2017, Argentina, Ecuador, and Uruguay reported similar proportions. Latin American countries are not the only ones with this problem, some European countries also attributed large proportions of deaths –up to two thirds - from uterine cancer to uterus, unspecified in 1960. However, this proportion decreased to less than a third in the 1990s [4]. In any case, the large majority of uterine cancer deaths below age 45 are likely due to cervical cancer.

## Strengths and limitations

This study has limitations such as missing data, and variation in death certification validity and completeness. In addition, we could not retrieve mortality data from Guatemala and Bolivia, two of the poorest countries in Latin America. Moreover, several countries evidenced variations of mortality rates during the study period, which can be explained by the quality of death registration in each country. The main strength of our study is the comprehensive description of recent trends and predicted mortality of cervical cancer in LAC countries. Our results provide timely information to policy makers regarding the current and future trends of cervical cancer, which could help in the development of public health interventions.

## Conclusions

Most of the LAC countries showed a decrease of mortality rates between 1997 and 2017. The variations of the mortality rates between countries during the last period of observation can be largely explained by the timing of implementation of nation-wide screening campaigns in different time periods and the variation of public health programs within each country, among others. Despite favorable downward trend, the mortality rate in some LAC countries remains high. HPV vaccination, screening, and early diagnosis and treatment are necessary to accelerate a rapid decline in cervical cancer mortality by 2030.

## Supplementary Information

**Additional file 1.**

## Data Availability

The datasets generated and/or analyzed during the current study was obtained from a publicly available: https://www-dep.iarc.fr/WHOdb/WHOdb.htm

## Abbreviations

LMICs: low- and middle-income countries
LAC: Latin America and the Caribbean
WHO: World Health Organization
HPV: Human papillomavirus
AAPC: Average Annual percent change
