# Supplementary material for: Cervical cancer mortality among young women in Latin America and the Caribbean: trend analysis from 1997 to 2030
Source: BMC Public Health. 2022 Jan 16;22:113. doi: 10.1186/s12889-021-12413-0 (PMC8761303; doi:10.1186/s12889-021-12413-0)

**Figure S1.** Age-standardized (world population) mortality rates for cervical cancer (C53 and including C54 [uterine cancer] and C55 [unspecified uterine cancer] in ICD-10) per 100,000 women aged 20−44 years in Latin American and Caribbean countries, in 2014−2017 (unless specified in parentheses).


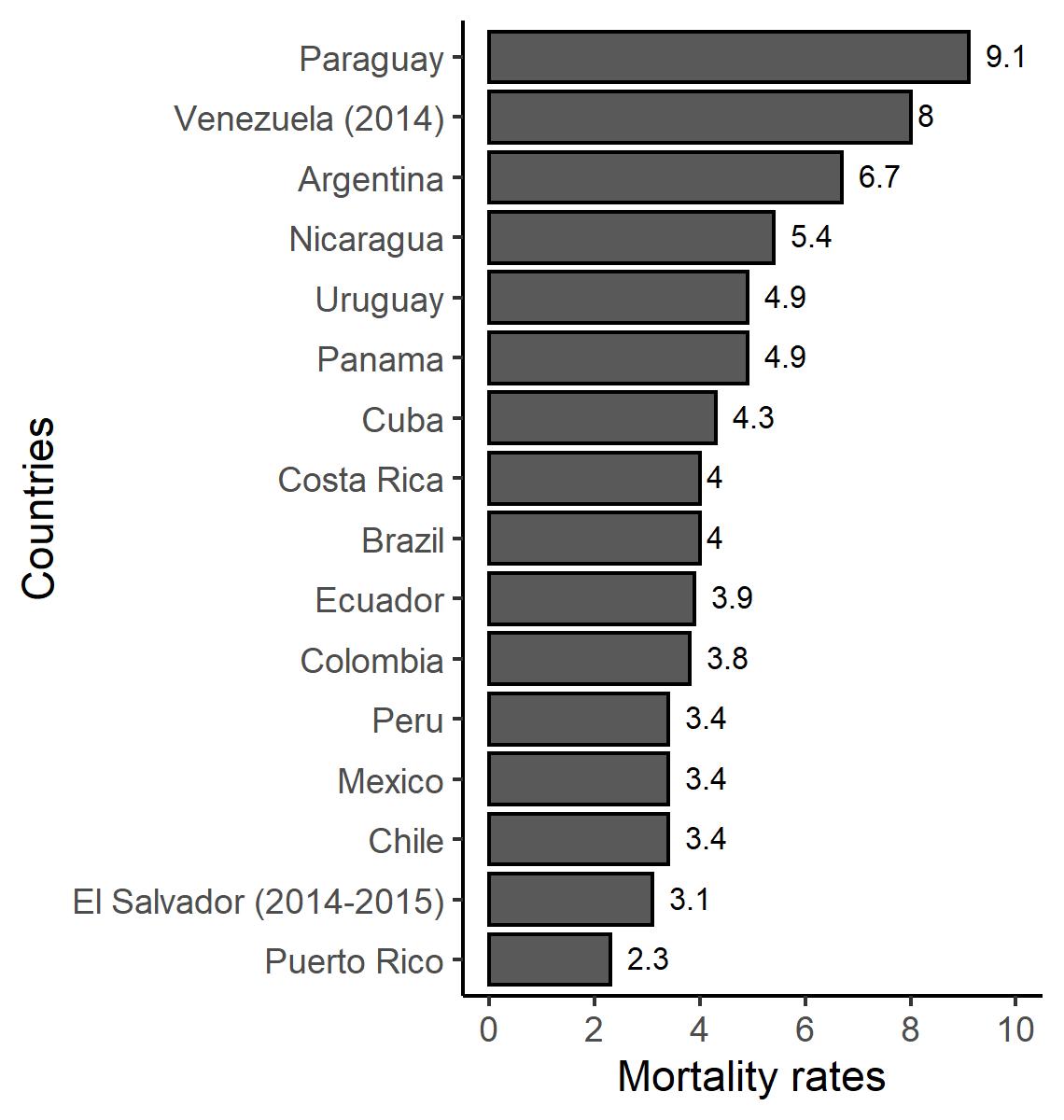


**Figure S2**. Age-standardized (world population) mortality rates of cervical cancer (C53 and including C54 and C55 in ICD-10) in Latin America and the Caribbean, 1997-2030


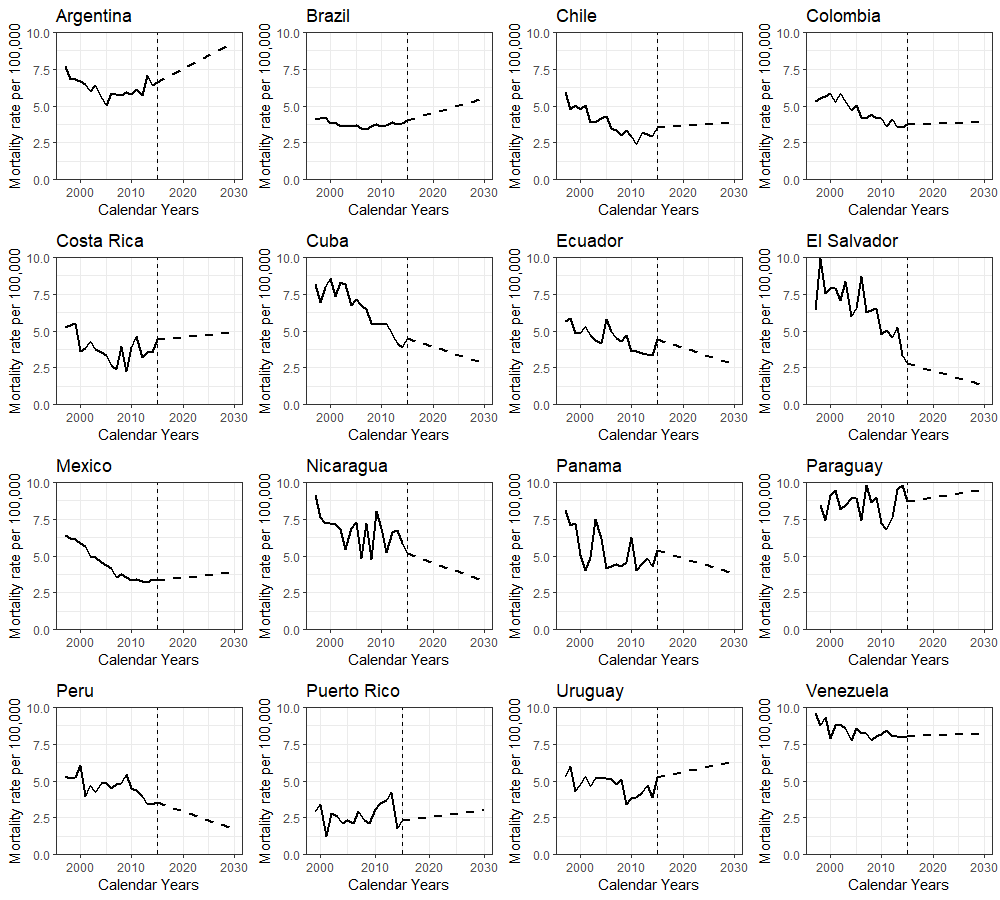


Table S1. Joinpoint analysis for cervical cancer mortality (C53 and including C54 and C55 in ICD-10) in Latin America and the Caribbean, 1997-2017

| **Countries** | **Year** | **APC** | **95% CI** | **Year** | **APC** | **95% CI** | **AAPC** | **95% CI** |
| --- | --- | --- | --- | --- | --- | --- | --- | --- |
| Argentina | 1997-2005 | −3.9* | −5.4, −2.4 | 2005−2017 | 2.1* | 1.2, 2.9 | -0.4 | −1.1, 0.4 |
| Brazil | 1997-2006 | −2.1* | −2.9, −1.3 | 2006−2017 | 1.7* | 1.1, 2.3 | 0.1 | −0.1, 0.2 |
| Chile | 1997-2011 | −4.8* | −5.9, −3.8 | 2011−2017 | 4.6* | 0.3, 9.1 | −2.1* | −3.3, −0.8 |
| Colombia | 1997-2017 | −2.4* | −3.0, −1.8 |  |  |  | −2.4* | −3.0, −1.8 |
| Costa Rica | 1997-2006 | −7.0* | −11.2, −2.5 | 2006−2017 | 3.5* | 0.1, 7.2 | −1.3 | −3.9, 1.3 |
| Cuba | 1997-2017 | −3.7* | −4.5, −3.0 |  |  |  | −3.7* | −4.5, −3.0 |
| Ecuador | 1997-2017 | −2.0* | −2.9, −1.2 |  |  |  | −2.0* | −2.9, −1.2 |
| El Salvador | 1997-2015 | −4.5* | −6.1, −2.9 |  |  |  | −4.5* | −6.1, −2.9 |
| Mexico | 1997−2017 | −3.6* | −4.3, −2.9 |  |  |  | −3.6* | −4.3, −2.9 |
| Nicaragua | 1997-2017 | −1.7* | −2.8, −0.6 |  |  |  | −1.7* | −2.8, −0.6 |
| Panama | 1997-2017 | −1.7* | −3.2, −0.2 |  |  |  | −1.7* | −3.2, −0.2 |
| Paraguay | 1997-2011 | −0.1 | −1.0, 0.8 |  |  |  | −0.1 | −1.0, 0.8 |
| Peru | 1997−2017 | −2.3* | −3.1, −1.4 |  |  |  | −2.3* | −3.1, −1.4 |
| Puerto Rico | 1999-2017 | 0.6 | −2.0, 3.3 |  |  |  | 0.6 | −2.0, 3.3 |
| Uruguay | 1997-2011 | −2.2* | −3.8, −0.5 | 2011−2017 | 4.6 | −1.7, 11.2 | −0.2 | −2.2, 1.9 |
| Venezuela | 1997-2014 | −0.7* | −1.2, −0.3 |  |  |  | −0.7* | −1.2, −0.3 |

* Significantly different from 0 (p < 0.05)

AAPC: average annual percent change; CI: confidence interval

Table S2. Number of cervical cancer deaths (C53 and including C54 and C55 in ICD-10), age-standardized mortality rates and percentage change in cases due to population and risk, 2015 and 2030.

| **Country** | Population (annual million) | | Number of deaths | | Age-standardized rate | | Total change (%) | Change due to population (%) | Change due to risk (%) |
| --- | --- | --- | --- | --- | --- | --- | --- | --- | --- |
|  | 2015 | 2030 | 2015 | 2030 | 2015 | 2030 |  |  |  |
| Argentina | 7.9 | 8.6 | 547 | 871 | 6.6 | 9.3 | 55.5 | 9.6 | 45.9 |
| Brazil | 41.3 | 40.5 | 1772 | 2534 | 4.0 | 5.3 | 37.8 | 4.2 | 33.6 |
| Chile | 3.3 | 3.3 | 125 | 150 | 3.6 | 3.8 | 29.5 | 8.8 | 20.7 |
| Colombia | 9.5 | 10.0 | 366 | 440 | 3.7 | 4.0 | 18.8 | 12.0 | [6.8](https://www.physio-pedia.com/Epidemiology,_Prevalence_and_Incidence) |
| Costa Rica | 0.9 | 1.0 | 41 | 55 | 4.4 | 4.8 | 52.5 | 20.9 | 31.6 |
| Cuba | 1.9 | 1.7 | 104 | 54 | 4.5 | 2.8 | −44.1 | −11.6 | −32.5 |
| Ecuador | 3.0 | 3.6 | 132 | 104 | 4.4 | 2.7 | −10.3 | 27.8 | −38.2 |
| El Salvador | 1.2 | 1.4 | 34 | 19 | 2.8 | 1.3 | −53.0 | 22.6 | −75.6 |
| Mexico | 25.0 | 26.5 | 857 | 1133 | 3.3 | 3.9 | 21.8 | 11.2 | 10.7 |
| Nicaragua | 1.2 | 1.4 | 62 | 50 | 5.2 | 3.2 | −26.0 | 27.0 | −53.9 |
| Panama | 0.8 | 0.9 | 42 | 57 | 5.4 | 3.8 | 53.0 | 95.4 | −42.5 |
| Paraguay | 1.2 | 1.5 | 98 | 153 | 8.7 | 9.5 | 50.9 | 46.0 | 4.9 |
| Peru | 6.1 | 6.6 | 215 | 126 | 3.6 | 1.7 | −38.4 | 21.1 | −59.6 |
| Puerto Rico | 0.7 | 0.5 | 17 | 14 | 2.3 | 2.6 | −29.5 | −36.8 | 7.3 |
| Uruguay | 0.6 | 0.6 | 34 | 42 | 5.3 | 6.2 | 34.1 | 4.8 | 29.3 |
| Venezuela | 5.8 | 6.3 | 463 | 549 | 8.0 | 8.2 | 18.7 | 12.8 | 5.9 |

**Figure S3**. Cervical cancer mortality rates (C53 and including C54 and C55 in ICD-10) in Latin American and Caribbean countries for 2015 and 2030, total change (change), change due to change in risk (risk), and change due to demographic alterations (population).


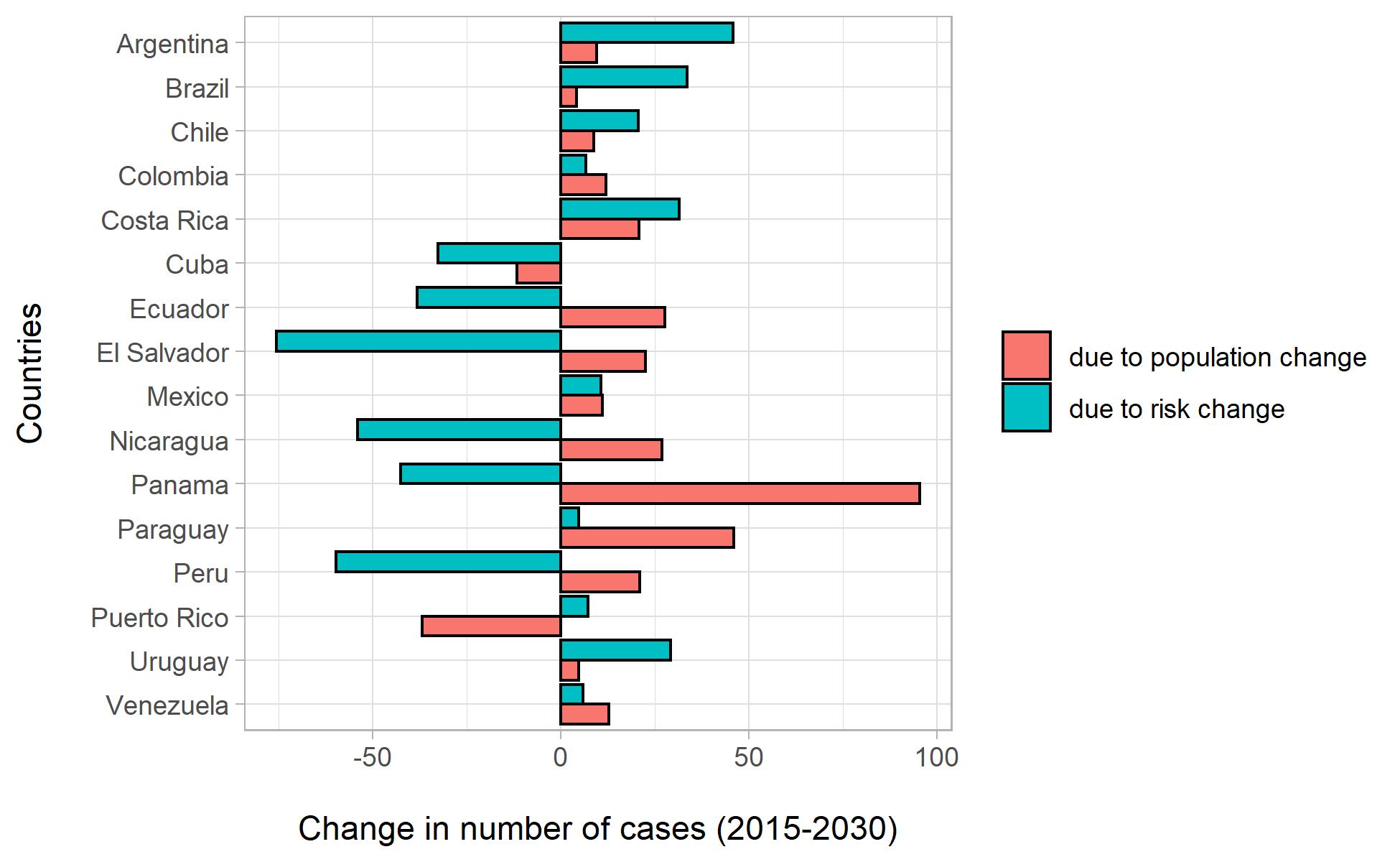

Supplement: Supplementary file 1 — Additional file 1. [file 12889_2021_12413_MOESM1_ESM.doc]
